# Impacts of school feeding on educational and health outcomes of school-age children and adolescents in low- and middle-income countries: protocol for a systematic review and meta-analysis

**DOI:** 10.1186/s13643-020-01317-6

**Published:** 2020-03-16

**Authors:** Dongqing Wang, Wafaie W. Fawzi

**Affiliations:** 1 Department of Global Health and Population, Harvard T.H. Chan School of Public Health, Harvard University, 90 Smith Street, 310 WS-5, Boston, MA 02120 USA; 2 Department of Epidemiology, Harvard T.H. Chan School of Public Health, Harvard University, Boston, MA USA; 3 Department of Nutrition, Harvard T.H. Chan School of Public Health, Harvard University, Boston, MA USA

**Keywords:** School feeding, School meal, Low- and middle-income countries, Children, Adolescents, Nutrition, Systematic review, Meta-analysis, Randomized controlled trials, Controlled before-after studies

## Abstract

**Background:**

School feeding programs are beneficial for the physical, mental, and psychosocial development of school-age children and adolescents, particularly those in low- and middle-income countries (LMICs). While school feeding programs are ubiquitous in LMICs, the specific benefits of school feeding programs are unclear. The aim of this systematic review and meta-analysis is to evaluate the impacts of school feeding programs on the educational and health outcomes of children and adolescents in LMICs.

**Methods:**

Rigorously designed interventional studies on the impacts of school feeding on nutritional and health outcomes of children and adolescents receiving primary or secondary education in LMICs will be included. The following information sources were used to identify relevant published or unpublished studies: MEDLINE, EMBASE, CINAHL, the Cochrane Library, and governmental or organizational websites. The risk of bias of randomized and non-randomized studies will be assessed using the Cochrane Risk of Bias tool and the ROBINS-I tool, respectively. Two reviewers will independently conduct the selection of studies, data extraction, and assessment of the risk of bias. A narrative synthesis of all the included studies will be provided. Meta-analyses will be performed whenever appropriate. Heterogeneity of effects will be assessed by *I*^2^, subgroup analyses, and meta-regression. The certainty of evidence for each outcome will be assessed using the Grading of Recommendation, Assessment, Development, and Evaluation (GRADE) approach.

**Discussion:**

The design and implementation of school feeding programs in LMICs should be based on the understanding of the benefits of such programs. This work will provide a crucial evidence base for the educational and health benefits of school feeding on children and adolescents in LMICs.

**Systematic review registration:**

This protocol was submitted for registration with the International Prospective Register of Systematic Reviews (PROSPERO) on November 18, 2019 (registration number: pending).

## Background

Nutrition during the school years is crucial for the physical, mental, and psychosocial development of children and adolescents aged 6 to 19 years. It is estimated that, across the developing world, 66 million school-age children go to school every day hungry, with 23 million hungry children in Africa [1]. Attending classes hungry severely impacts children’s and adolescents’ abilities to learn, to thrive, and to realize their full potentials [2].

School feeding programs (sometimes referred to as school meal programs) are interventions that regularly provide nutritious foods to children and adolescents attending school [3]. Benefits of school feeding on children and adolescents include alleviating hunger, reducing micronutrient deficiency and anemia, preventing overweight and obesity, improving school enrollment and attendance, increasing cognitive and academic performance, and contributing to gender equity in access to education [4–8]. Most countries have some forms of school feeding programs in some way and at some scale [6, 8]. School feeding programs are widely available in high-income countries but generally have incomplete coverages in low- and middle-income countries (LMICs), where the need is greatest in terms of hunger and poverty [5]. Most countries in sub-Saharan Africa only have school feeding interventions that are targeted toward the most food-insecure regions instead of being universally available [5]. It is imperative to expand the coverage of school feeding programs and to improve the quality of existing programs to maximize their benefits on children and adolescents.

Little is known about the impacts of school feeding programs on specific educational and health outcomes of school-age children and adolescents in LMICs. Previous reviews on the potential effects of school feeding are outdated, with the most recent Cochrane review published in 2007 [9], thus do not reflect all of the currently available evidence. Also, previous work has limited scopes in terms of the age range [10] or the outcomes examined (e.g., anthropometric and nutritional outcomes but not educational or psychosocial outcomes or vice versa) [11]. Further, prior reviews have focused on the *provision* of school meals and did not explicitly evaluate what specific *content* (types and amounts of foods and nutrients) of the school meals conferred the largest benefits on outcomes [9]. Therefore, an updated and refined synthesis of evidence on school feeding interventions and a wide range of educational and health outcomes of children and adolescents is warranted and will inform the design and implementation of future programs.

The aim of this systematic review and meta-analysis is to evaluate the impacts of school feeding programs on educational and health outcomes of children and adolescents aged 6 to 19 receiving primary or secondary education in LMICs. We will emphasize findings generated from randomized controlled trials (RCTs). RCTs better account for external factors that may confound the effect of school feeding programs, including background nutritional deficiency levels and inputs from schools and teachers [8, 12]. We will also include other rigorously designed interventional studies, including controlled before-after studies (CBAs) and non-randomized controlled trials that were able to account for the baseline differences between intervention arms [9].

## Methods/design

### Research question

We aim to evaluate the impacts of school feeding programs on educational and health outcomes of children and adolescents receiving primary or secondary education in LMICs. We also aim to assess the potentially different impacts of school feeding by characteristics of the program and by composition of the foods provided.

### Eligibility criteria

#### Inclusion criteria

1. We will include RCTs, with the intervention randomized individually or in clusters (classes or schools). We will also include CBAs as they are non-randomized studies with a relatively rigorous design and occupy a non-negligible proportion of the relevant literature [9]. Non-randomized controlled trials are also eligible for inclusion as long as the baseline differences between intervention arms were accounted for in the analysis.
2. We will include published articles as well as unpublished and grey literature and will include ongoing studies where preliminary findings are available to us.
3. Studies conducted in LMICs as defined by the World Bank 2020 fiscal year.
4. Studies involving children and adolescents (boys and girls) aged 6 to 19 who were receiving primary or secondary education (i.e., primary, middle, or high school).
5. Studies that examined the impacts of the provision of foods, including meals (breakfast, lunch, or dinner) or snacks consumed at school (in-school feeding), and foods distributed to the family and consumed outside of the school setting (take-home ration) [5]. We will consider the provision of solid foods or beverages (e.g., milk). We will also include studies that examined food stamps or food vouchers distributed at school for the participants to access foods (in the market or food banks).
6. The comparison (control) group in each included study can be participants who did not receive school feeding or any other interventions, or participants who received alternative interventions instead of school feeding. We will also consider the comparison of school feeding programs with different food compositions, such as the comparison between an updated program with an original one.
7. We will include educational, nutritional, anthropometric, cognitive, and morbidity outcomes of children and adolescents. Potential outcomes include height, weight, skinfold thickness, mid-upper arm circumference, micronutrient status, hemoglobin level, school enrollment, school attendance, dropout, school achievement (math, reading, spelling), on-task behavior, cognition, and morbidity (e.g., fever, cough, diarrhea, and vomiting). Studies with results for at least one outcome of interest will be included.
8. No restrictions will be placed on the year, language, sample size of the study, or the duration of the intervention.

#### Exclusion criteria

1. Non-randomized controlled trials that did not account for the baseline differences between intervention arms.
2. Interventional studies without a proper control group, such as uncontrolled before-after studies, uncontrolled interrupted time series studies, and uncontrolled difference-in-difference designs.
3. Observational studies (e.g., cohort, case-control, and cross-sectional studies).
4. Editorials, commentaries, opinions, and review articles (these will, however, be used to identify additional original studies).
5. Studies conducted among preschool children only. Feeding interventions among preschool children are important and of great interest but are beyond the scope of this work, which will focus on the school setting.
6. Studies that examined the impacts of micronutrient fortification, micronutrient supplementation, or nutrition education; however, if such interventions are complementary to otherwise eligible school feeding interventions, these studies will be included.
7. Clinical treatment programs targeted toward individuals with specific medical conditions, or programs toward underweight, overweight, or obese individuals.
8. Studies that only examined aggregate-level economical or agricultural outcomes.
9. Studies that described school feeding programs without linkage to specific outcomes.

### Information sources

The following databases were searched for eligible studies, from the inception of each database through November 2019: MEDLINE (via PubMed), EMBASE, CINAHL, and the Cochrane Library. The selection of the four electronic databases was made in consultation with a health science librarian with expertise in systematic searching. Our search covered the three databases (i.e., MEDLINE, EMBASE, and the Cochrane Library) that are recommended by the *Cochrane Handbook for Systematic Reviews of Interventions* [13]. We also searched ClinicalTrials.gov and other governmental or organizational websites (World Food Programme (WFP), World Health Organization, Food and Agriculture Organization (FAO), and World Bank) for studies not identified from the database searching. We will conduct a manual search of references of retained articles and previous reviews. We will also consult with content experts on school feeding to identify any additional studies. Reports written in languages other than English will be translated by colleagues who are native speakers of the corresponding languages whenever possible. Studies that cannot be adequately translated will be excluded.

### Search strategy

We consulted with a health science librarian to develop the PubMed search strategy, which is provided in Additional File 1. The sensitivity of the search strategy was examined by confirming that several sentinel articles were identified. The PubMed strategy will be adapted to the syntax appropriate for other databases. The initial search took place in November 2019, and an updated search will be conducted in early April 2020.

### Data management

EndNote X9 (Clarivate Analytics, PA, USA) will be used to store the records retrieved from searches of electronic databases. The records will also be imported into Covidence (Veritas Health Innovation, Melbourne, Australia), an internet-based program that facilitates the streamlined management of the systematic review. Duplicate records will be detected and removed first by EndNote and then by Covidence.

### Selection of studies

The results of the searches will be independently assessed by two reviewers based on the inclusion and exclusion criteria. All titles and abstracts will be reviewed first to remove irrelevant studies. For potentially eligible studies and studies with unclear eligibility, the full texts will be obtained and reviewed to confirm eligibility using a form for full text screening, which will be pilot tested on five randomly selected full texts. Disagreements between reviewers will be resolved by discussion or by a third reviewer when necessary. Inter-rater agreement will be quantified by calculating the raw percentage of agreement and Cohen’s *κ* coefficient. Specific reasons for study exclusions will be documented and summarized using the flow diagram for the Preferred Reporting Items for Systematic Reviews and Meta-Analyses (PRISMA) [14]. Neither of the reviewers will be blind to journal titles or the names of the authors.

### Data extraction

Data of the retained studies will be extracted by two reviewers independently and entered into a data extraction form, which will be pilot tested on five randomly selected studies. Disagreements in the extracted information between reviewers will be resolved by discussion or by a third reviewer. When necessary, the corresponding authors of the studies will be contacted to obtain relevant information. We will extract the following information: title, authors (first author and corresponding author), contact information of corresponding author, journal (or source for unpublished reports), calendar year of publication, calendar year of intervention, country, source of funding, study design, sample size (number of clusters for each group and number of participants in each group), sample characteristics (e.g., age, sex, and socioeconomic status), intervention (including timing, duration, food and nutritional content, and co-interventions), measure of adherence, comparator/control, outcomes assessed, main findings with point estimates and measures of variance (standard errors, 95% confidence intervals, or *p* values), theory to explain success (if available), and theory to explain failure (if available). Multiple reports of a single study will be collated as additional results may be provided in different reports. Whenever there are inconsistent results across reports of a single study, we will contact the corresponding author to obtain more accurate results. The data extraction form is provided in Additional File 2.

### Assessment of risk of bias

The risk of bias will be independently assessed by two reviewers. Any disagreement on the risk of bias between reviewers will be resolved by discussion and by a third reviewer when necessary. The risk of bias assessments will be conducted for each outcome reported in each trial, rather than for the whole study. For RCTs, we will use version 2 of the Cochrane Risk of Bias tool (RoB 2) [15], which considers the following five domains: bias arising from the randomization process, bias due to deviations from intended interventions, bias due to missing outcome data, bias in measurement of the outcome, and bias in selection of the reported results. For cluster-randomized trials, we will additionally consider bias from the timing of identification and recruitment of individual participants in relation to timing of randomization [16]. Each domain will be judged as “low risk of bias,” “high risk of bias,” or “some concerns.” We will consider an RCT to be of low risk of bias if it is judged to have low risk of bias for all domains; we will consider an RCT to be of high risk of bias if it is judged to have high risk of bias in at least one domain or have some concerns for multiple domains (≥ 3) in a way that substantially lowers confidence in the result; we will consider an RCT to have some concerns if it raises some concerns in at least one domain but not to be at high risk of bias for any domain [15]. For CBAs and non-randomized controlled trials, we will use the Risk of Bias in Non-randomized Studies of Interventions (ROBINS-I) tool [17], which considers biases from confounding, bias in selection of participants into the study, bias in classification of interventions, bias due to deviations from intended interventions, bias due to missing data, bias in measurement of outcomes, and bias in selection of the reported results. Each domain will be judged as “low risk of bias,” “moderate risk of bias,” “serious risk of bias,” “critical risk of bias,” or “no information.” We will consider a non-randomized study to be of low risk of bias if it is judged to have low or moderate risk of bias for all domains; we will consider a non-randomized study to be of high risk of bias if it is judged to have serious or critical risk of bias in one or more domains; we will consider a non-randomized study to have some concerns if the assessment is unclear for one or more domains but low or moderate for all other domains. We will contact the corresponding authors of the reports to obtain more information when necessary. We will summarize the results of the assessment of the risk of bias in a table, in which the judgment for each domain will be presented with a justification [15].

### Data synthesis

A systematic and narrative synthesis of all included studies will be presented in the text and also as a table. School feeding will be treated as a dichotomous exposure (i.e., intervention vs. control). Effect estimates for continuous outcomes will be expressed as mean differences (with 95% confidence intervals) comparing the intervention group with the control group; effect estimates for dichotomous outcomes will be expressed as risk ratios, rate ratios, hazard ratios, or odds ratios (all with 95% confidence intervals), comparing the intervention group with the control group. For RCTs, we will extract the results based on intention-to-treat analyses. When more than two intervention groups are present in a study, they will be treated as separate arms. However, when the interventions of the additional arms are not relevant to school feeding, they will not be taken into account. Ideally, cluster-randomized studies should report results from analyses that appropriately account for the study design, such as mixed-effects models or generalized estimating equations. Studies that ignored clustering are overprecise and will receive unduly high weights in the meta-analysis. When cluster-based studies did not use the proper statistical methods to account for clustering, we will extract or apply an intraclass correlation coefficient to modify the standard errors based on the approach described in the *Cochrane Handbook for Systematic Reviews of Interventions* [13].

If studies for a given outcome are sufficiently consistent in terms of intervention, comparator, and outcome definition, we will conduct a random-effects, inverse-variance-weighted meta-analysis for the outcome. The random-effects method will be used as the effect of school feeding is presumed to be heterogeneous across time and populations. Heterogeneity of effects across studies will be assessed by computing the *I*^2^ statistic, which represents the percentage of the total variation in the effect estimates that is due to true heterogeneity rather than chance; *I*^2^ > 50% will be considered as substantial heterogeneity [18].

We will assess the sources of heterogeneity by conducting subgroup analyses with the following prespecified characteristics: unit of allocation (individual, class, or school), modality of intervention (in-school meal, in-school snacks, take-home ration, food stamps/food vouchers), presence of co-interventions (by itself or combined with complementary interventions), timing of intervention (breakfast, lunch, dinner, or snack), duration of intervention (defined as the interval between the initialization of the school feeding intervention, and when the outcomes were assessed), year of study, country or region, level of food insecurity of the region, age group of participants (primary or secondary education), sex of participants, type of report (published or unpublished), and risk of bias (low, high, or some concerns). To assess the potentially differential impacts by the specific content of the meal, we will conduct exploratory subgroup analyses by the type and amount of the foods or nutrients entailed in the program, such as the presence of fruits, vegetables, and animal source foods, or the adequacy of micronutrients and macronutrients (defined in relation to the Recommended Dietary Allowances). To further explain heterogeneity, we will perform meta-regression using the predictors mentioned above. We will use contour-enhanced funnel plots to detect publication bias if there are 10 or more studies available for an outcome [19]. We will assess the robustness of the results by excluding studies judged to have a high risk of bias, and by repeating the analyses using fixed-effects models. We will compute for each outcome a 95% prediction interval, which provides a predicted range of the effect of the intervention when applied in an individual setting [20]. Statistical analyses will be conducted using STATA 16 (StataCorp, College Station, Texas). For outcomes with insufficient data or extreme heterogeneity that cannot be assessed in subgroup analyses or meta-regression, we will provide a narrative synthesis without a meta-analysis.

### Assessment of certainty of evidence

The overall certainty of evidence for each included outcome will be assessed using the Grading of Recommendation, Assessment, Development, and Evaluation (GRADE) approach, which considers risk of bias, publication bias, imprecision, inconsistency, and indirectness [21–26]. The strength of the overall evidence will be judged as high, moderate, low, or very low [21].

### Registration and reporting

This protocol was submitted for registration with the International Prospective Register of Systematic Reviews (PROSPERO) on November 18, 2019. However, at the time of this proofing, the protocol has still not been reviewed or assigned a registration number. We reached out to PROSPERO for an update on the registration status and was told that the registration for non-U.K. studies would take a long time (4-5 months with a minimum of 140 days). In the event of protocol amendments, the date of each amendment will be accompanied by a description of each change and the rationale on PROSPERO. We prepared this protocol following the Preferred Reporting Items for Systematic Review and Meta-Analysis Protocols (PRISMA-P) [27]. The PRISMA-P checklist can be obtained from Additional File 3. We will report this systematic review following the *Cochrane Handbook for Systematic Reviews of Interventions* [13] and the PRISMA guidelines [14].

## Discussion

School feeding programs have been and will continue to be essential for the provision of nutrients, improvement of academic performance, and the promotion of a healthy lifestyle in LMICs. Therefore, there is a strong political will to continue to fund new programs and to expand on existing programs [28]. The design and implementation of school feeding programs in LMICs should be based on the established benefits of such programs on specific educational and health outcomes of children and adolescents, for which an updated evidence base is needed.

*The State of School Feeding Worldwide*, published by the WFP, summarized the status of school feeding across the world and reported that school feeding programs are ubiquitously present. However, the quality of school feeding programs varies greatly across countries and with national income [6]. The report also highlighted a need to strengthen the evidence base on the potential benefits of school feeding. Drake et al. reviewed the design and implementation of 14 school feeding programs in LMICs [28]. They concluded that there is no one-size-fits-all model for school feeding programs, given that different countries approach school feeding programs with different objectives. However, they did identify some good practices that are likely applicable across countries, such as the inclusion of fruits and vegetables, the collaboration with local smallholder farmers, and the incorporation of school feeding programs as the component of a much broader curriculum of nutrition and health education. They noted that there was a lack of quantitative data on the impacts of school feeding, especially those from randomized controlled trials. A recently published report by the FAO-reviewed nutrition guidelines and standards for school meals from 33 LMICs through surveys targeted toward relevant stakeholders and found considerable variation between and within countries in terms of coordination, management, funding, objectives, and modalities of school feeding programs [3]. For example, the objectives of the identified school feeding programs include addressing short-term hunger, reducing nutrient deficiency, improving attendance and school performance, encouraging healthy eating habits, and supporting local agriculture and economy. Lunch is the most common timing for the identified programs, and the majority of the school lunch programs offer cooked meals that range from single dishes based on staples with added vegetables, legumes, and animal-source foods to menus with a main dish and a side dish; fruits are provided as part of the meal in some programs. Nutrition education and school gardens (often also for educational purposes) are the most common complementary interventions to the feeding programs. However, this report focused on government-owned programs and excluded any pilot projects or scalable programs coordinated by non-governmental entities. None of the reports mentioned above quantitatively linked school feeding programs to specific outcomes of children and adolescents.

Numerous systematic reviews have synthesized the impacts of school-based dietary interventions on various outcomes of children and adolescents [9–11, 29–45]. However, most of the reviews consist primarily of nutrition education programs that did not provide actual foods. To date, only a few reviews focused on or were able to draw conclusions regarding school feeding programs [9–11, 44, 45]. Kristjansson et al. conducted the first systematic review on this topic with 18 studies (nine from lower-income countries) among socio-economically disadvantaged children and adolescents across the world [9]. It was reported that, in RCTs from lower-income countries, participants who were fed at school gained an average of 0.39 kg more weight over 19 months and attended school more frequently (4 to 6 more days per year per participant) than those in control groups; school-fed participants in lower-income countries also had better performances in math and short-term cognitive tasks compared to controls. However, results for height and results from higher-income countries are mixed, suggesting that the benefit of school feeding varies by outcome and by socioeconomic status, with disadvantaged participants from lower-income countries likely to benefit more from school feeding. While comprehensive at the time, this first systematic review only included seven RCTs, five of which were conducted in lower-income countries. Jomaa et al. reviewed the impacts of school feeding programs on educational and health outcomes of primary-school-age children in developing countries. They reported relatively consistent positive associations between school feeding and energy intake, micronutrient status, school enrollment, and school attendance, but reported inconclusive results on growth, cognition, and academic achievement [10]. However, this review did not include children and adolescents at the secondary school level or studies conducted prior to 1990. Krishnaratne et al. reviewed rigorously designed studies among children and adolescents in LMICs. They found significant associations between school feeding and enrollment, dropout, progression, and nonsignificant associations with attendance and learning [44]. Snilstveit et al. systematically reviewed interventions for improving learning outcomes and access to education for children and adolescents in LMICs and reported positive associations between school feeding and enrollment, attendance, and various learning outcomes [45]. The reviews by Krishnaratne [44] and Snilstveit [45], however, did not specifically focus on school feeding, nor did they consider non-educational endpoints such as nutrition and health. Watkins et al. reviewed the impacts of school feeding on the nutritional status of primary-school-age children and preschool and adolescent girls in LMICs; they reported small and significant effects on weight gain and small and nonsignificant effects on height gain among school-age children [11]. Nevertheless, this review focused on anthropometric outcomes and nutritional status and had limited coverage on educational or psychosocial outcomes. None of the previous reviews examined in detail whether different *content* (e.g., types and amounts of foods and nutrients) of the provided meals had differential impacts on children and adolescents, which is crucial information for the design and improvement of future programs.

School years represent a critical period not only for physical and mental development but also for the formation of long-term dietary and lifestyle habits. This systematic review and meta-analysis will provide a comprehensive evidence base for the development and refinement of future school feeding programs targeted toward children and adolescents in LMICs.

## Supplementary information

**Additional File 1.** PubMed search strategy.

**Additional File 2.** Data extraction form.

**Additional File 3.** PRISMA-P 2015 Checklist.

## Data Availability

All data that will be generated and analyzed during this study will be included in the published article or its supplementary information files.

## Abbreviations

CBA: Controlled before-after study
FAO: Food and Agriculture Organization
GRADE: Grading of Recommendation, Assessment, Development, and Evaluation
LMIC: Low- and middle-income country
PRISMA: Preferred Reporting Items for Systematic Reviews and Meta-Analyses
PRISMA-P: Preferred Reporting Items for Systematic Review and Meta-Analysis Protocols
PROSPERO: International Prospective Register of Systematic Reviews
RCT: Randomized controlled trial
ROBINS-I: Risk of Bias in Non-randomized Studies of Interventions
WFP: World Food Programme
