# Supplementary material for: Impacts of school feeding on educational and health outcomes of school-age children and adolescents in low- and middle-income countries: protocol for a systematic review and meta-analysis
Source: Syst Rev. 2020 Mar 16;9:55. doi: 10.1186/s13643-020-01317-6 (PMC7075040; doi:10.1186/s13643-020-01317-6)
Supplement: Supplementary file 1 — Additional File 1. PubMed search strategy. [file 13643_2020_1317_MOESM1_ESM.docx]

|  | **Additional File 1**  **PubMed search strategy** | |  |
| --- | --- | --- | --- |
| **No.** | **Concept** | **PubMed search terms** | **Number of records**  **(As of November 12, 2019)** |
| #1 | Randomized controlled trial | randomized controlled trial[pt] OR "randomized controlled trials as topic"[MeSH] OR “controlled trial”[tiab] OR “intervention*”[tiab] OR "random allocation"[MeSH] OR random*[tiab] OR trial*[tiab] | 2,213,419 |
| #2 | Controlled before-after studies | "controlled before-after studies"[MeSH] OR CBA[tiab] OR CBAs[tiab] OR “before-after”[tiab] OR “before after”[tiab] OR “before-and-after”[tiab] OR “before and after”[tiab] | 244,320 |
| #3 | School feeding | ("schools"[MeSH] OR school*[tiab] OR "School Health Services"[Mesh]) AND (("meals"[MeSH] OR meal*[tiab]) OR ("lunch"[MeSH] OR lunch*[tiab]) OR ("breakfast"[MeSH] OR breakfast*[tiab]) OR dinner*[tiab] OR ("snacks"[MeSH] OR snack*[tiab]) OR biscuit[tiab] OR ("diet"[MeSH] OR diet[tiab] OR diets[tiab] OR dietary[tiab]) OR ("eating"[MeSH] OR eating[tiab]) OR ("food"[MeSH] OR food*[tiab]) OR (“beverages"[MeSH] OR beverage*[tiab]) OR feed*[tiab] OR "diet, food, and nutrition"[MeSH] OR cater*[tiab] OR cafe*[tiab] OR ("fruit"[MeSH] OR fruit*[tiab]) OR ("vegetables"[MeSH] OR vegetable*[tiab]) OR ("meat"[MeSH] OR meat*[tiab]) OR ("milk"[MeSH] OR milk[tiab]) OR nutritio*[tiab] OR menu*[tiab] OR canteen*[tiab] OR (“gardens”[MeSH] or garden*[tiab]) OR ("food assistance"[MeSH] OR stamp*[tiab] OR voucher*[tiab])) | 37,814 |
| #4 | Children or adolescents | “child”[MeSH] OR “students”[MeSH] OR “adolescent”[MeSH] OR child[tiab] OR children[tiab] OR schoolchildren[tiab] OR kids[tiab] OR kid[tiab] OR student*[tiab] OR teen*[tiab] OR adolescen*[tiab] OR preteen*[tiab] | 3,519,775 |
| #5 | Low- and middle-income countries | "Developing Countries"[MeSH] OR developing countr*[tiab] OR developing nation*[tiab] OR less developed countr*[tiab] OR less developed nation*[tiab] OR third world nation*[tiab] OR third world countr*[tiab] OR under developed nation*[tiab] OR underdeveloped nation*[tiab] OR under developed countr*[tiab] OR underdeveloped countr*[tiab] OR middle income countr*[tiab] OR middle income nation*[tiab] OR low income countr*[tiab] OR low income nation*[tiab] OR poor countr*[tiab] OR poor nation*[tiab] OR lmic[tiab] OR lmics[tiab] OR "Africa"[MeSH] OR "Asia"[MeSH] OR "South America"[MeSH] OR "Latin America"[MeSH] OR "Central America"[MeSH] OR africa[tiab] OR asia[tiab] OR south america*[tiab] OR latin america*[tiab] OR central america*[tiab] OR Afghanistan*[tiab] OR Albania*[tiab] OR Algeria*[tiab] OR Samoa*[tiab] OR Angola*[tiab] OR Armenia*[tiab] OR Azerbaijan*[tiab] OR Bangladesh*[tiab] OR Bengali[tiab] OR Belarus*[tiab] OR Belize[tiab] OR Benin[tiab] OR Bhutan*[tiab] OR Bolivia*[tiab] OR Bosnia*[tiab] OR Herzegovina*[tiab] OR Botswana*[tiab] OR Brazil*[tiab] OR Bulgaria*[tiab] OR Burkina Faso[tiab] OR Burundi*[tiab] OR Cabo Verd*[tiab] OR Cape Verd*[tiab] OR Cambodia*[tiab] OR Cameroon*[tiab] OR Central African*[tiab] OR Chad*[tiab] OR China[tiab] OR Chinese[tiab] OR Colombia*[tiab] OR Comoros[tiab] OR Congo[tiab] OR Cook Islands[tiab] OR Costa Rica*[tiab] OR Cote d'Ivoire[tiab] OR Ivory Coast[tiab] OR Cuba[tiab] OR Cuban[tiab] OR Djibouti[tiab] OR Dominica*[tiab] OR Ecuador[tiab] OR Egypt[tiab] OR El Salvador*[tiab] OR Eritrea*[tiab] OR Ethiopia*[tiab] OR Falkland Islands[tiab] OR Fiji*[tiab] OR Gabon*[tiab] OR Gambia*[tiab] OR Georgia*[tiab] OR Ghana*[tiab] OR Grenada*[tiab] OR Guadeloupe[tiab] OR Guatemala*[tiab] OR Guian*[tiab] OR Guinea*[tiab] OR Guyan*[tiab] OR Haiti*[tiab] OR Hondura*[tiab] OR India[tiab] OR Indian*[tiab] OR Indonesia*[tiab] OR Iran*[tiab] OR Iraq*[tiab] OR Jamaica*[tiab] OR Jordan*[tiab] OR Kazakh*[tiab] OR Kenya*[tiab] OR Kiribati[tiab] OR People's Republic of Korea[tiab] OR North Korea[tiab] OR Kosovo[tiab] OR Kosovar*[tiab] OR Kyrgyz*[tiab] OR Lao[tiab] OR Laos[tiab] OR Laotian*[tiab] OR Lebanon[tiab] OR Lebanes*[tiab] OR Lesotho[tiab] OR Liberia*[tiab] OR Libya*[tiab] OR Macedonia*[tiab] OR Madagascar*[tiab] OR Malawi*[tiab] OR Malvinas[tiab] OR Malaysia*[tiab] OR Maldives[tiab] OR Mali[tiab] OR Marshall Island*[tiab] OR Mauritania*[tiab] OR Mauriti*[tiab] OR Mayotte[tiab] OR Mexico[MeSH] OR Mexican*[tiab] OR Micronesia*[tiab] OR Moldova*[tiab] OR Mongolia*[tiab] OR Montenegr*[tiab] OR Montserrat[tiab] OR Morocc*[tiab] OR Mozambique[tiab] OR Myanmar[tiab] OR Burmese*[tiab] OR Burma[tiab] OR Namibia*[tiab] OR Nauru[tiab] OR Nepal*[tiab] OR Netherlands Antilles[tiab] OR Nicaragua*[tiab] OR Niger*[tiab] OR Niue[tiab] OR Pakistan*[tiab] OR Paraguay*[tiab] OR Peru*[tiab] OR Philippin*[tiab] OR Pitcairn[tiab] OR Romania*[tiab] OR Rwanda*[tiab] OR Sao Tome[tiab] OR Principe[tiab] OR Senegal*[tiab] OR Serbia*[tiab] OR Sierra Leone*[tiab] OR Solomon Island*[tiab] OR Somalia*[tiab] OR South Africa*[tiab] OR Sri Lanka[tiab] OR St Helena[tiab] OR Saint Helena[tiab] OR St Lucia[tiab] OR Saint Lucia[tiab] OR St Vincent[tiab] OR Saint Vincent[tiab] OR Grenad*[tiab] OR Sudan*[tiab] OR Suriname*[tiab] OR Swaziland*[tiab] OR Eswatini*[tiab] OR Syria*[tiab] OR Tajik*[tiab] OR Tanzania*[tiab] OR Thai*[tiab] OR Timor*[tiab] OR Togo*[tiab] OR Tokelau[tiab] OR Tonga*[tiab] OR Tunisia*[tiab] OR Turkey[tiab] OR Turkish[tiab] OR Turkmen*[tiab] OR Tuvalu*[tiab] OR Uganda*[tiab] OR Ukrain*[tiab] OR Uzbeki*[tiab] OR Vanuatu*[tiab] OR Venezuela*[tiab] OR Vietnam*[tiab] OR Viet nam*[tiab] OR West Bank[tiab] OR Gaza*[tiab] OR Palestin*[tiab] OR Wallis and Futuna[tiab] OR Yemen*[tiab] OR Zambia*[tiab] OR Zimbabw*[tiab] OR Western Sahara[tiab] OR Argentin*[tiab] OR Russia*[tiab] | 2,034,763 |
| RCTs | #1 AND #3 AND #4 AND #5 |  | 2,859 |
| CBAs | #2 AND #3 AND #4 AND #5 |  | 166 |
| Total | (#1 OR #2) AND #3 AND #4 AND #5 |  | 2,921 |

Abbreviations: CBA, controlled before-after studies; RCT, randomized controlled trial.
