# Supplementary material for: Impacts of school feeding on educational and health outcomes of school-age children and adolescents in low- and middle-income countries: protocol for a systematic review and meta-analysis
Source: Syst Rev. 2020 Mar 16;9:55. doi: 10.1186/s13643-020-01317-6 (PMC7075040; doi:10.1186/s13643-020-01317-6)
Supplement: Supplementary file 2 — Additional File 2. Data extraction form. [file 13643_2020_1317_MOESM2_ESM.docx]

**Additional File 2**

**Data extraction form**

| **Title** | **Author** | **Journal or source** | **Year of publication** | **Year of study** | **Country** | **Source of funding** | **Study design** | **Sample size** | **Sample characteristics (age, sex, socioeconomic status, etc.)** | **Timing of intervention** | **Duration of intervention** | **Content of intervention** | **Adherence** | **Control group** | **Outcomes** | **Main findings** | **Theory to explain success** | **Theory to explain failure** |
| --- | --- | --- | --- | --- | --- | --- | --- | --- | --- | --- | --- | --- | --- | --- | --- | --- | --- | --- |
|  |  |  |  |  |  |  |  |  |  |  |  |  |  |  |  |  |  |  |
|  |  |  |  |  |  |  |  |  |  |  |  |  |  |  |  |  |  |  |
|  |  |  |  |  |  |  |  |  |  |  |  |  |  |  |  |  |  |  |
|  |  |  |  |  |  |  |  |  |  |  |  |  |  |  |  |  |  |  |
|  |  |  |  |  |  |  |  |  |  |  |  |  |  |  |  |  |  |  |
|  |  |  |  |  |  |  |  |  |  |  |  |  |  |  |  |  |  |  |
|  |  |  |  |  |  |  |  |  |  |  |  |  |  |  |  |  |  |  |
